# The Impact of Caregiver Affiliate Stigma on the Psychosocial Well-Being of Caregivers of Individuals with Neurodegenerative Disorders: A Scoping Review

**DOI:** 10.3390/healthcare12191957

**Published:** 2024-10-01

**Authors:** Nelly Becerra Carrillo, Massimo Guasconi, Serena Barello

**Affiliations:** 1 Behavioural Health Psychology Lab, Department of Brain and Behavioural Sciences, University of Pavia, 27100 Pavia, Italy; nellydelcarmen.becerracarrill01@universitadipavia.it; 2 Department of Medicine and Surgery, University of Parma, 43121 Parma, Italy; massimo.guasconi@unipr.it; 3 Azienda USL of Piacenza, 29121 Piacanza, Italy; 4 IRCCS National Institute of Neurology, C. Mondino Foundation, 27100 Pavia, Italy

**Keywords:** affiliate stigma, stigma, neurodegenerative disorders, ecological systems theory, psychosocial well-being, scoping review

## Abstract

**Background.** Caregiver affiliate stigma concerning neurodegenerative disorders (NDDs) profoundly affects caregivers’ well-being, though its full impact remains to be fully elucidated. **Objectives.** This scoping review aims to consolidate the current knowledge on caregiver affiliate stigma related to NDDs, explore its connection to caregiver psychosocial well-being, and pinpoint the gaps in the existing research. **Methods**. Adhering to the PRISMA-ScR guidelines, a comprehensive search of five databases was conducted for peer-reviewed, English language studies focusing on caregiver-affiliated stigma in relation to NDDs. **Results**. The initial search yielded 9033 articles, with 19 meeting the inclusion criteria after rigorous screening. Bronfenbrenner’s ecological systems theory was employed to analyze various stigma conceptualizations, including public, courtesy, affiliate, and family stigma. Analysis revealed a correlation between elevated levels of affiliate stigma and increased caregiver burden, deteriorated mental health outcomes, and diminished quality of life. The internalization of stigma was found to vary across demographic groups, influenced by factors such as education level and social support. The review also highlighted the mediating role of coping strategies and the protective function of social support against stigma internalization. **Conclusions**, These findings underscore the necessity for targeted, culturally sensitive interventions that address caregiver affiliate stigma across multiple ecological levels. This review contributes to a more nuanced understanding of caregiver affiliate stigma in relation to NDDs, laying the groundwork for future research and intervention development aimed at enhancing caregiver well-being in diverse cultural contexts.

## 1. Introduction

Global population demographics are shifting toward an aging population. Projections indicate that, by 2050, individuals over 65 years old will comprise 16% of the global population, doubling the number of children under the age of five years. This translates to an estimated increase from 771 million people over 65 years old in 2022 to 1.6 billion by 2050 [1]. Accompanying this demographic shift is a rise in the prevalence of neurodegenerative disorders (NDDs), such as Alzheimer’s disease (AD), Parkinson’s disease (PD), and motor neuron diseases, which are among the most common [2,3]. These diseases are characterized by the progressive loss of neuronal functions and are becoming more prevalent with time [3].

NDDs impact multiple aspects of human functioning, often limiting individuals’ ability to perform basic tasks. These disorders are chronic and typically incurable, leading to a long-term dependence on caregivers [4]. Currently, over 55 million people worldwide live with dementia, and approximately 10 million new cases are reported annually, positioning NDDs as a significant public health concern [5]. The most common form of dementia is AD, which is characterized by the progressive accumulation of β-amyloid plaques and tau tangles, leading to a cognitive decline in and impairment of memory, reasoning, and language skills [6,7]. PD, the second most common NDD [8], is marked by tremors, muscle stiffness, and movement difficulties, resulting from the degeneration of dopaminergic neurons in the substantia nigra [9]. Recent research suggests a more complex progression of the disease, potentially starting years before the motor symptoms appear [10]. Similarly, Amyotrophic Lateral Sclerosis (ALS), a progressive motor neuron disease, affects motor neurons in the brain and spinal cord, leading to muscle degeneration and paralysis [11]. While most cases of ALS are sporadic, around 5–10% are inherited [12]

The impact of these disorders extends beyond individuals, heavily burdening caregivers who play a crucial role in managing the daily lives of those with NDDs [13,14]. The complexity of NDDs comes from the complex interaction of genetic, epigenetic, and environmental factors [15]. Caregiving for individuals with NDDs is associated with substantial psychological stress, often exceeding physical strain [16]. Caregivers’ mental and physical health, personal resources, and available social support critically influence their ability to cope with the demands of caregiving [16,17,18]. The nature and intensity of challenges experienced by caregivers vary based on the disease, but high levels of depression and burden are common, with the research showing depression rates exceeding 30% among caregivers of AD patients [19,20,21]. Key predictors for depressive symptoms include caregivers’ health, personal resources, and perceived adequacy in their caregiving roles [22].

Furthermore, the severity of problematic behaviors exhibited by individuals with dementia, especially in long-term care settings, is strongly correlated with poorer mental and physical health outcomes for caregivers [23]. Despite these challenges, caregiving can also be a source of personal growth and fulfilment. Many caregivers report experiencing a sense of purpose, increased self-worth, and strengthened relationships through their caregiving role [24,25,26]. This complex interplay between the challenges and rewards of caregiving emphasizes the need to consider caregivers’ quality of life (QoL) as a critical outcome.

QoL, as described by Felce and Perry [27], encompasses both objective conditions and subjective evaluations of well-being across physical, social, emotional, and material domains. For caregivers of individuals with NDDs, QoL can be significantly affected by the progression of the disorder. The research suggests a link between declining QoL in caregivers and increasing disease severity in care recipients, highlighting the dynamic interaction between caregiver well-being and patient health [28].

A particularly challenging aspect of caregiving for individuals with NDDs is the phenomenon of stigma. Stigma, defined by Andersen et al. [29], refers to societal labeling, stereotyping, and negative judgments. Caregiver affiliate stigma, the focus of this review, refers to the internalization of these societal biases by caregivers, leading to negative self-perceptions and social withdrawal [30,31]. Various types of stigma, such as public stigma, courtesy stigma (stigma by association), and family stigma, all contribute to caregivers’ psychological burden. Public stigma involves negative societal reactions toward individuals with NDDs, while courtesy stigma extends this discrimination to caregivers due to their association with stigmatized individuals [32,33]. Family stigma specifically addresses the negative experiences of family members linked to a relative with a stigmatized condition [34].

The measurement of caregiver affiliate stigma has employed tools such as the Affiliate Stigma Scale (ASS) [30], alongside broader burden measures, like the Caregiver Burden Inventory (CBI) [35] and Zarit Burden Interview (ZBI) [36]. Instruments designed to assess depression and anxiety are also commonly used to understand the psychological impact of stigma on caregivers [37,38]. While the research on caregiver affiliate stigma is growing, it remains fragmented. Although scales like the ASS and the Family Stigma in Alzheimer’s Disease Scale (FS-ADS) [39] provide some standardization, further work is needed to refine definitions and enhance the validity of these measures across different contexts.

Despite an increased awareness of caregiver affiliate stigma, the research findings have yet to be fully translated into effective policies and interventions. While some countries have incorporated caregiver support into their dementia strategies, as seen in the World Health Organization’s Global Action Plan on the Public Response to Dementia (2017–2025) [40], stigma-related interventions remain limited. Current interventions primarily focus on general stigma reduction, such as psychoeducational programs and support groups, rather than addressing the unique needs of caregivers [41]. Without a comprehensive understanding of how caregiver affiliate stigma affects well-being, it is difficult to develop targeted strategies to support caregivers.

Therefore, this review aims to systematically map the definitions, measures, and interventions related to caregiver affiliate stigma to better understand its impact on caregiver well-being and identify pathways for effective intervention.

### Aims and Objectives

This scoping review aims to comprehensively map the existing literature on the impact of caregiver affiliate stigma of caregivers for individuals with NDDs, on their psychosocial well-being. More specifically this review aimed to: (1) Examine the relationship between affiliate stigma and caregiver psychosocial well-being; (2) Identify and synthesize existing definitions and conceptualizations of caregiver affiliate stigma and related concepts within the context of NDDs through the lenses of Bronfenbrenner’s ecological systems theory; and (3) Identify the knowledge gaps in the existing literature, highlighting areas where further investigation is needed. Finally, using the Bronfenbrenner’s ecological system theory, we discuss the review results to identify possible lines of interventions to prevent caregiver affiliate stigma phenomena.

## 2. Materials and Methods

### 2.1. Study Design

A scoping review methodology was adopted, guided by the methodological framework outlined by Arksey and O’Malley [42], and further developed by Peters et al. [43] in nine key stages: (1) Defining and aligning the objective/s and question/s; (2) Developing and aligning the inclusion criteria with the objective/s and question/s; (3) Describing the planned approach; (4) Searching for evidence; (5) Selecting the evidence; (6) Extracting the evidence; (7) Analysis of the evidence; (8) Presentation of the results; and (9) Summarizing the evidence. Moreover, this scoping review adhered to the PRISMA-ScR guidelines [44] to ensure a systematic and comprehensive approach. 

### 2.2. Eligibility Criteria

The review included peer-reviewed studies published in English, with no time limitations, which focused on primary caregivers of individuals diagnosed with any NDDs. Studies were required to be quantitative and to address caregiver-affiliate stigma in the context of NDDs and its impact on psychological well-being, including, but not limited to, the concepts of affiliate stigma, courtesy stigma, or stigma by association, family stigma, and caregiver stigma. The inclusion criteria specified the use of validated measures of stigma and psychosocial well-being, as well as the reported quantitative outcomes related to the impact of stigma on caregiver well-being. 

Studies were excluded if they did not specifically address caregiver-affiliate stigma or the related concepts mentioned in the inclusion criteria above, focused solely on the stigma experienced by individuals with NDDs, or lacked outcomes related to psychosocial well-being.

### 2.3. Types of Sources 

The search strategy included electronic databases (PubMed/MEDLINE, PsycINFO, Embase, CINAHL, and Scopus), gray literature sources (Google Scholar, ProQuest Dissertations and Theses, and relevant organizational websites), and reference lists of included studies and relevant reviews.

### 2.4. Search Strategy 

A comprehensive search was performed across five electronic databases: Pubmed, Embase, CINAHL, PsycInfo, and Scopus. The search strategy incorporated keywords and MeSH terms related to caregiver affiliate stigma, neurodegenerative disorders, and psychosocial well-being. Boolean operators were utilized to link search terms within and between concepts. Supplementary file S1 (Table S1) illustrates the search string applied in the used databases. Comparable strategies, tailored to the specific syntax and requirements of each platform, were employed for the remaining databases.

### 2.5. Source of Evidence Selection

Two independent reviewers (NBC and SB) screened titles and abstracts for relevance using Rayyan.ai, a web and mobile app for systematic reviews. The platform’s collaborative features allowed for efficient conflict resolution and progress tracking. Full-text articles were assessed against the eligibility criteria. Any discrepancies were resolved through discussions between the two reviewers or, when necessary, through a consultation with a third reviewer (MG). 

**Figure 1:**
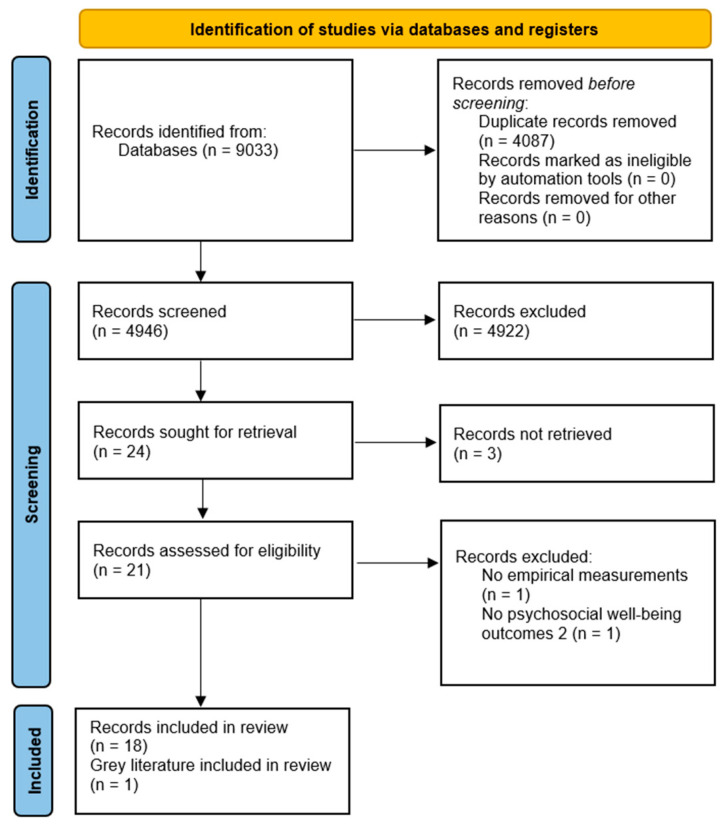
PRISMA flow diagram for identification, screening, eligibility, and inclusion.

The study selection process, including the number of studies identified, screened, assessed for eligibility, and included in the final analysis, is illustrated in the PRISMA flow diagram (Figure 1).

### 2.6. Data Extraction

Data were extracted using a standardized form, including study characteristics (e.g., author, year, and study design), participant characteristics, stigma measurement tools and definitions, outcomes related to caregiver psychosocial well-being, and key findings (Table 1).

### 2.7. Data Synthesis and Analysis 

The findings were synthesized with a narrative approach, guided by Bronfenbrenner’s ecological system theory. Bronfenbrenner’s model proposes four interconnected systems influencing human development and behavior: microsystem, mesosystem, exosystem, and macrosystem [45]. This framework was applied to classify and analyze different types of stigma identified in the literature. 

The synthesis focused on mapping the conceptualizations of stigma on these ecological levels, providing a structured understanding of caregiver affiliate stigma in relation to NDDs. This theoretical framework was chosen due to its capacity to elucidate the multiple levels at which stigma operated, ranging from individual experiences to broader societal attitudes. This approach allowed for a more nuanced understanding of how different forms of stigma interact and influence caregiver well-being, potentially informing more comprehensive and effective interventions. 

This scoping review was registered to the Open Science Framework (OSF) on 27 August 2024, with the following DOI: https://doi.org/10.17605/OSF.IO/MD5JE.

**Table 1:** Characteristics of included studies on caregiver affiliate stigma in relation to neurodegenerative disorders.

Author and Year of Publication	Country of Study	Study Population	Sample Size	Aim of the Study	Study Design	Instruments Used	Type of Stigma and Definition
Bhatt et al., 2022 [46]	United Kingdom	Family carers of individuals with primary progressive dementia	70	To investigate the validity of the Family Stigma Instrument (FAMSI) and use it to explore the extent to which experiences of stigma are endorsed in relation to family carers of people living with dementia	Cross-sectional	FAMSI	Stigma by association or courtesy stigma: refers to the stigma directed toward individuals because of their association with a stigmatized person
						Rosenberg Self-Esteem Scale (RSES)	
						Demographics Questionnaire	Affiliate stigma: involves the internalization of stigma by association
Brundige, 2022 [47]	United States	Husband/male long-term cohabitating life-partner caregivers of women with AD	72	To examine whether high gender role conflict and stigma by association in husband/male life-partner caregivers of women with Alzheimer’s disease are significantly related to their vulnerability to self-isolation	Cross-sectional	Geriatric Depression Scale (GDS)	Stigma by association: the prejudice and discrimination that are extended to people not because of some mark that they manifest, but rather because they are somehow linked to a person with the stigmatized mark
						Bem Sex Role Inventory (BSRI)	
						Family Stigma in Alzheimer’s Disease Scale-Caregiver (FS-ADS-C) scale	
						Marwit–Meuser Caregiver Grief Inventory Worry and Felt Isolation (MMCGI-WFI) subscale	
						Multilevel Assessment Instrument—Social Interaction (MAI-SI) subscale	
Chang et al., 2016 [48]	Taiwan	Caregivers of family members diagnosed with dementia	271	To examine the psychometric properties of ASS when used with caregivers of family members diagnosed with dementia	Cross-sectional	ASS	Structural stigma: the imbalances and injustices in social structures
						CBI	Public stigma: the negative reactions from the general population toward a stigmatized group
						Taiwanese Depression Questionnaire (TDQ)	Self-stigma: internalization of public stigma
						28-item World Health Organization Quality of Life Questionnaire (WHOQOL-BREF)	Courtesy stigma: prejudice and discrimination, which are extended to people due to their relationship to a person with a stigmatized mark
						Beck Anxiety Inventory (BAI)	Affiliate stigma: the internalization of courtesy stigma
Chen et al., 2023 [49]	Taiwan	Dyads of people with dementia and their informal caregivers	261	To investigate the mediating roles of caregiver burden and affiliate stigma in the relationship between neuropsychiatric symptoms of people with dementia and the mental health outcomes (depression and anxiety) of their caregivers	Cross-sectional	CBI	Affiliate stigma: for internalized stigma
						ASS	
						TDQ	
						BAI	Courtesy stigma: caregivers become stigmatized because of their family member's mental illness
						Neuropsychiatric Inventory (NPI)	
Ellin et al., 2023 [50]	Malaysia	Caregivers of patients with dementia	178	To assess the impact of affiliate stigma on the psychological well-being of caregivers of patients with dementia	Cross-sectional	ASS	Caregiver stigma: negative perceptions and stigma among caregivers. It can be classified as associative or affiliate stigma
						Psychological Well-Being (PWB)	Associative stigma: stigmatization of a family member due to the association with the patient
							Affiliate stigma: known as self-stigma
Hu et al., 2023 [51]	Taiwan	Family caregivers of individuals with dementia	275	To explore the associations between affiliate stigma, caregiver burden, psychological distress, and QoL among family caregivers of people with dementia	Cross-sectional	CBI	Affiliate stigma: type of stigma with features of courtesy stigma and self-stigma
						WHOQOL-BREF	
						ASS	Courtesy stigma: suffering from stigma due to the connection, association, or relationship with a stigmatized group
						BAI	
						TDQ	Self-stigma: endorsing and internalizing stigma within oneself
Jeong et al., 2020 [52]	South Korea	Family caregivers of individuals with Alzheimer’s or other forms of dementia	226	To investigate the relationship between family caregivers, examining whether this relationship is mediated by a caregiver’s enhanced coping efficacy and moderated by a caregiver’s affiliate stigma	Cross-sectional	Information Cross-Checking	Affiliate stigma: internalized stigma
						Modified Coping Efficacy Scale	
						ASS	Courtesy stigma: stigma from social association with a stigmatized individual
						Modified Physical Coping Outcome Scale	
Liu et al., 2014 [53]	United States	Caregivers of persons with dementia in the early stages of the disease	51	To examine the relationship between perceived stigma and depressive symptoms among caregivers of persons with dementia	Longitudinal	Mini Mental State Examination (MMSE),	Perceived stigma: the labeling behaviors of others, which creates an internalization process and results in negative consequences
						Clinical Dementia Rating Scale (CDR),	
						Knowledge of Alzheimer’s Test Family Version (FKAT),	
						Revised Memory and Behavior Problems Checklist (RMBPC),	Courtesy stigma: family members experience stigma due to their association with persons with a mental illness or dementia
						Adapted Stigma Impact Scale (SIS),	
						and Center for Epidemiologic Studies Depression Scale (CES-D)	
Saffari et al., 2018 [54]	Iran	Primary caregiver of older adults with dementia	664	To examine if and how spiritual coping and stigma-related family stress impact the associations between the patient's activities of daily living impairments and caregiver mental health	Longitudinal	ZBI	Social stigma: social stigmatization directed toward a person due to their neurological condition
						Spiritual Coping Strategies (SCSs)	
						Lawton Instrumental Activities of Daily Living (IADL) scale	
						Short Form 12 (SF-12)	
						MMSE	Family stigma: extension of social stigma to the family
						Hospital Anxiety and Depression Scale (HADS)	
						Family Stigma Stress Scale (FSSS)	
Saffari et al., 2019 [38]	Iran	Caregivers of older adults with dementia	541	To establish the psychometric properties of ASS among Iranian caregivers of people with dementia	Cross-sectional	ASS	Courtesy stigma: the individual is affiliated with a stigmatized group
						ZBI	
						HADS	Public stigma: the negative reactions from society toward stigmatized people.
						SF-12	
						RSES	Affiliate stigma: when the negative reactions are internalized
						Multidimensional Scale of Perceived Social Support (MSPSS)	Family stigma: when the caregiver is a family member
Sommers-Spijkerman et al., 2023 [55]	The Netherlands	Caregivers of patients diagnosed with Amyotrophic Lateral Sclerosis (ALS) or Progressive Muscular Atrophy (PMA)	87	To investigate the experiences of stigma among ALS/PMA patients and their caregivers, and to identify potential associated factors of stigma	Cross-sectional	Stigma Scale for Chronic Illness (SSCI)	Enacted stigma: refers to the actual discrimination, prejudice, and negative behaviors that individuals with a disease and their caregivers experience from others
						Amyotrophic Lateral Sclerosis Functional Rating Scale—Revised (ALS-FRS-R)	Felt stigma: refers to the internalization of societal attitudes by the individuals with the disease and their caregivers, leading them to feel shame, embarrassment, or a fear of discrimination
						ASS	Affiliate stigma: stigma experienced by the caregiver due to their association with the stigmatized person
Su and Chang, 2020 [56]	Taiwan	Caregivers of a family member aged older than 65 years with any type of dementia	270	To investigate the relationship between caregiver burden in family caregivers of a person with dementia and affiliate stigma, as well as the demographic and clinical factors contributing to this stigma type	Cross-sectional	CBI	Affiliate stigma: internalization of negative societal views
						ASS	
						TDQ	
						BAI	
						NPI	Courtesy stigma or stigma by association: involves the negative behaviors from the public toward caregivers, relatives, and health professionals associated with the patient
						Barthel Index (BI)	
						Clinical Dementia Rating (CDR)	
						MMSE	
Tudose et al., 2017 [57]	Romania	Family members of patients admitted to a psychiatric hospital with the diagnosis of dementia	76	To investigate the relationship between perceived stigma, expressed emotion (EE), and QoL among caregivers of individuals with dementia	Cross-sectional	Patient and caregiver demographics questionnaire	Structural stigma: the imbalances and injustices in social structures, political decisions, and legal regulations
						ASS	
						FS-ADS	Affiliate stigma: internalized public stigma
						Involvement Evaluation Questionnaire (IEQ)	
Van den Bossche and Schoenmakers, 2022 [58]	Belgium	Relatives of patients with a formal diagnosis of dementia	228	To determine the impact of affiliate stigma on the mental well-being of relatives caring for a person with dementia	Cross-sectional	ASS	Courtesy stigma: discrimination and prejudice that people may experience because they are associated with individuals associated with a stigmatized group
						Items of Patient Health Questionnaire-9 (PHQ-9)	
						Items of the 20-item CES-D	Affiliate stigma: negative feelings that relatives of stigmatized individuals develop toward themselves because they perceive the associative stigma that prevails in society
Velilla et al., 2022 [59]	Colombia	Caregivers of patients with early-onset Alzheimer’s disease due to E280A mutation in presenilin 1 (EOAD), frontotemporal dementia (FTD), and late-onset Alzheimer’s disease (LOAD)	151	To assess the impacts of family stigma and socioeconomic factors on psychological outcomes, QoL, and caregiver burden on caregivers of patients with early-onset AD	Cross-sectional	Structured interview about socioeconomic factors	Self-stigma: when individuals accept and internalize the stigma
						ASS	
						Functional Assessment Staging (FAST)	
						Frontal Behavioral Inventory (FBI)	Courtesy stigma: when prejudice and discrimination extend from stigmatized people to their friends or relatives who do not present marks of the stigmatized condition
						ZBI	
						CES-D,	Family stigma: courtesy stigma experienced by family caregivers
						Spielberger State-Trait Personal Inventory (STPI), and	
						36-Item Short Form Survey (SF-36)	
Weisman de Mamani et al., 2017 [60]	United States	Caregivers of individuals with dementia	106	To examine the relationship between stigma, EE, and QoL in caregivers of individuals with age-related dementia	Cross-sectional	CDR,	Perceived stigma: caregiver’s perceptions of negative attitudes and behaviors directed toward them by others due to their role of caring for individuals with dementia
						20-item Family Questionnaire (FQ),	
						Quality of Life Inventory (QOLI), and	
						Modified SIS	
Werner and AboJabel, 2020 [61]	Israel	Israeli Arab family caregivers of persons with dementia	175	To examine the characteristics of family caregivers of persons with dementia who internalize courtesy stigma, and to investigate the process of this internalization	Cross-sectional	Authors-developed courtesy and Affiliate Stigma Scale	Courtesy stigma: caregiver’s perceptions of public stereotypes about the person with the stigmatic condition
						Activities of Daily Living (ADL) scale	
						IADL	Affiliate stigma: self-stigma experienced by the caregivers of the stigmatized person
						Cognitive Status Scale	
						Problematic Behaviour Scale	
						MSPSS	
						Cope Multidimensional Coping Inventory Short Scale (MCI)	Family stigma: associated with providing care for a relative
						Zarit Burden Interview Short Form (ZBI-SF)	
Werner et al., 2011 [39]	Israel	Children of people with Alzheimer’s Disease (AD)	185	To develop and examine the validity of a scale specifically designed to measure family stigma associated with AD	Cross-sectional	FS-ADS	Structural stigma: the social aspect of stigma
							Caregiver stigma: intrapersonal aspect of stigma by association
							Public stigma: reactions of people toward a stigmatized individual or group
						ZBI-SF	Courtesy stigma/stigma by association: emotions and beliefs of those who surround the stigmatized person
						Problematic Behavior Scale	Family stigma: perception of stigma that comes from being associated with a relative with AD
							Self-stigma: internalization of ideas and the reactions of the people personally targeted by a stigma
Werner et al., 2012 [34]	Israel	Adult child caregivers of elderly parents diagnosed with probable AD	185	To examine whether family stigma is a predictor of caregiver burden in the context of AD	Cross-sectional	ZBI-SF	Public stigma: perceptions and reactions of the general public toward both the person targeted by stigma
						FS-ADS	Self-stigma: internalization of the ideas and reactions of those personally targeted by stigma
						Problematic Behavior Scale	Courtesy stigma: the emotions and beliefs of those surrounding the stigmatized person

## 3. Results

As illustrated in the PRISMA flow diagram (Figure 1), the initial database search resulted in 9033 articles across the five databases (Scopus: 3490; PubMed: 3098; Embase: 1182; CINAHL: 643; PsycInfo: 620). After removing duplicated and screening titles and abstracts, 24 full-text articles were assessed for eligibility. Of these, 19 met the inclusion criteria and were included in the final analysis.

### 3.1. Study Characteristics

The included 19 studies represented a diverse geographical distribution, encompassing multiple countries (Taiwan (n = 4), Israel (n = 3), United States (n = 3), and Iran (n = 2)) and one study each from Malaysia, South Korea, Colombia, the Netherlands, Belgium, Romania, and the United Kingdom. This geographical diversity provides a broad perspective on caregiver-affiliate stigma across different cultural contexts.

Study designs were predominantly cross-sectional (n = 17), with a minority of longitudinal studies (n = 2). Sample sizes varied considerably, ranging from 51 to 664 participants, with a median sample size of 185.

The majority of articles (n = 16) focused on caregivers of people with various types of dementia. A smaller number of studies specifically examined caregivers of those with AD only (n = 2) and one study included caregivers of patients with ALS or PMA. Notably, despite Parkinson’s disease being the second most common NDD [8], none of the 19 studies included in this review specifically focused on caregivers of individuals with Parkinson’s disease. This represents a significant gap in the current literature on caregiver affiliate stigma in relation to NDDs.

### 3.2. Impacts of Caregiver Affiliate Stigma on Well-Being and Determinants

Table 2 presents a summary of the key findings from the included research, demonstrating the varied effects of stigma on the caregiver’s well-being. Consistently across the studies, higher levels of caregiver-affiliate stigma were associated with poorer outcomes for caregivers, specifically:

- Mental health: multiple studies reported significant correlations between affiliate stigma and increased symptoms of depression and anxiety in caregivers [48,49].
- Quality of life: higher levels of stigma were linked to lower QoL scores [51].
- Caregiver burden: studies consistently found a positive association between affiliate stigma and caregiver burden [34,56].

### 3.3. Factors Influencing Stigma Internalization

The internalization of stigma varied among demographic groups and was influenced by several factors:

- Educational level: Werner and Abojabel [61] found that lower education was associated with higher levels of affiliate stigma.
- Social support: social support emerged as an important protective factor against stigma internalization [61].
- Gender: Van den Bossche and Schoenmakers [58] reported that women experienced greater impacts of affiliate stigma on their mental well-being. However, Su and Chang [56] found that male caregivers experienced higher levels of anxiety and care burden related to affiliate stigma compared to females.
- Relationship to care recipient: Werner et al. [34] reported that adult children experienced lower levels of stigma compared to other caregivers.
- Age and duration of caregiving: Van den Bossche and Schoenmakers [58] found that a longer duration of dementia diagnosis and older caregiver age were associated with higher affiliate stigma.

### 3.4. Conceptualization and Measurement of Stigma 

The analysis revealed varied conceptualizations of stigma related to NDD caregivers. Eleven distinct constructs were identified across the studies: courtesy stigma or stigma by association (n = 15), affiliate stigma (n = 12), family stigma (n = 4), public stigma (n = 4), self-stigma (n = 4), structural stigma (n = 3), perceived stigma (n = 2), caregiver stigma (n = 2), social stigma (n = 1), enacted stigma (n = 1), and felt stigma (n = 1).

Several validated instruments were used to measure these constructs; the ASS (n = 11) was most frequently employed, demonstrating good psychometric properties across different cultural contexts [38,48]. Other commonly used instruments included the BAI (n = 4), CBI, (n = 4), TDW (n = 4), CES-D (n = 3), FS-ADS (n = 3), MMSE (n = 3), ZBI (n = 3), and various versions of the ZBI (n = 6).

### 3.5. Terminological Inconsistencies in Relation to Stigma Conceptualization

Analysis of the included studies revealed a notable lack of consensus in the terminology used to describe internalized stigma among caregivers of individuals with NDDs. The inconsistency highlights the complex nature of stigma in the caregiving context.

Affiliate stigma, employed by Chang et al. [48], Su and Chang [56], and Hu et al. [51] was frequently used to describe the internalization of public stigma by caregivers. Chen et al. [49] utilized the phrase self-stigma to refer to a similar process of internalizing negative stereotypes, while Sommers Spijkerman et al. [55] used the phrase felt stigma. These concepts appear to overlap with affiliate stigma, though the authors used distinct terminology. 

Werner et al. [39] introduced the concept of family stigma, specifically addressing the stigma experienced by family caregivers of individuals with Alzheimer’s disease. This phrase aims to capture the unique dynamics of stigma within the family context of NDD caregiving. Similarly, the phrase caregiver stigma used by Ellin et al. [50], which includes both internalized and perceived stigma experienced by caregivers.

Courtesy stigma and stigma by association are used interchangeably across the included literature. For instance, Bhatt et al. [46] and Brundige [47] both used these phrases to describe stigma directed toward individuals due to their association with a stigmatized person.

Other phrases were used, like public stigma and structural stigma, across studies to describe different aspects of the stigma experience [57,61].

This variability in terminology underscores the need for a more standardized approach to the conceptualization and measuring of caregiver-affiliate stigma in relation to NDDs.

For a comprehensive overview of the terminology variations and conceptualizations of stigma across the reviewed studies, please refer to Table 1.

### 3.6. Ecological Systems’ Analysis of Stigma

**Figure 2:**
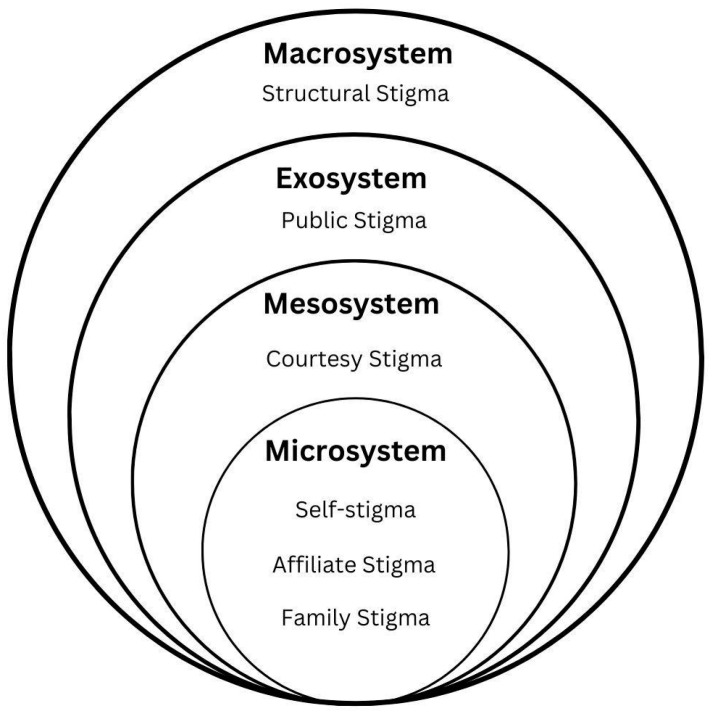
Ecological system classification of stigma in relation to neurodegenerative disorders caregiving.

The use of Bronfenbrenner’s ecological system theory [45] for the findings, as shown in Figure 2, reveals how caregiver affiliate stigma operates across multiple social levels.

#### 3.6.1. Microsystem

At the innermost level, there are factors that directly impact the self-perception and daily experiences of caregivers. When talking about the internalization of stigma, four types of stigma were identified: self-stigma, affiliate stigma, family stigma, and caregiver stigma. Affiliate stigma, self-stigma, and family stigma were the most prominent concepts in the literature, warranting their inclusion at this level. While initially considered, the phrase caregiver stigma was ultimately excluded due to its ambiguous nature, potentially referring to both the stigma directed toward caregivers and the internalization of such a stigma [50]. This overgeneralization could perpetuate conceptual confusion and hinder a precise analysis.

#### 3.6.2. Mesosystem

The mesosystem refers to the interactions between different microsystems. The impact of the person being stigmatized due to their association with an NDD is reflected in the isolation of other groups by other individuals and/or themselves. This comes from their association with a stigmatized person, thus resulting in the position of courtesy stigma in this section. Werner and Abojabel [61] identified how factors such as family dynamics and social support network influence the internalization of stigma. Having a strong social support network acts as a buffer against the negative effects of stigma.

#### 3.6.3. Exosystem

At this broader level, it is crucial to explore public stigma. Van den Bossche and Schoenmakers [58] revealed that the impact of affiliate stigma varies among demographic groups, with women and the partners of those with dementia feeling more affected. These findings highlight how broader societal attitudes indirectly influence individual experiences of stigma. 

#### 3.6.4. Macrosystem

The macrosystem represents cultural attitudes and policies, and therefore the classification of structural stigma at this level. Tudose et al. [57] talked about how societal-level factors contribute to caregiver burden and affect their QoL. Their results showed how cultural norms and healthcare policies shape the overall context in which caregivers experience and cope with stigma. 

#### 3.6.5. Interaction across Systems

The classification of stigma types using Bronfenbrenner’s framework becomes useful to understand how stigma permeates broad cultural norms and personal beliefs. 

Starting at the macrosystem level, cultural values and societal beliefs about NDDs shape institutional practices and policies, influencing how healthcare systems and social services address the needs of individuals with NDDs and their caregivers. The exosystem serves as a conduit of these broad cultural attitudes into a more localized context. Here, public stigma is observed in community settings, workplaces, and healthcare systems, indirectly affecting caregivers even when they are not directly involved.

The mesosystem acts as a critical intermediary, where family dynamics, social networks, and immediate community interactions amplify the effects of broader stigma. The attitudes previously directed toward an individual with NDDs start to affect the caregivers. Finally, the microsystem level presents the culmination of these influences in the form of the internalization of the stigmatizing attitudes encountered by caregivers at the other levels. 

The permeability between these systems is the key to understanding the complex nature of stigma. Attitudes and beliefs do not simply flow from top–down. There is a constant feedback loop, where individuals’ experiences can, over time, influence broader societal views.

### 3.7. Cultural Variations in Stigma Experiences

Studies conducted in diverse cultural settings revealed both similarities and differences in stigma experiences. For example, Saffari et al. [54] found that spiritual coping strategies played a significant role in mediating the relationship between stigma and caregiver outcomes in an Iranian context, highlighting the importance of culturally sensitive approaches to understanding caregiver affiliate stigma. In contrast, Jeong et al. [52] focused on information seeking and efficacy as coping mechanisms when talking about the outcomes for caregivers with low affiliate stigma. Taiwan presented a unique gender dynamic [56], reporting higher levels of anxiety and care burden related to affiliate stigma among male caregivers, differing from Malaysia where Ellin et al. [50] associated the female gender with higher affiliate stigma. The Netherlands offered a broader perspective, with Sommers-Spijkerman et al. [55] reporting on both the enacted and felt stigma experienced by patients and caregivers alike, a distinction not prominently featured in studies from other countries. These findings illustrate the importance of considering the cultural context when understanding and addressing caregiver affiliate stigma in relation to NDDs.

## 4. Discussion

This scoping review synthesized the current knowledge on caregiver affiliate stigma in the context of neurodevelopmental disorders (NDDs), providing valuable insights into its conceptualization, measurement, and impact on caregiver well-being. The analysis of 19 studies revealed significant variability in the terminology and measurement of stigma, underscoring the complex and multifaceted nature of this phenomenon.

Higher levels of caregiver affiliate stigma were consistently associated with poorer outcomes for caregivers, including increased depression, anxiety, lower quality of life, and higher caregiver burden. These findings align with the broader literature on stigma in healthcare, such as mental health caregiving [62], while also revealing the unique challenges faced by NDD caregivers. For instance, the work of Werner and AboJabel [61] on Israeli Arab caregivers highlights the importance of considering cultural nuances when addressing stigma. This underscores the need for culturally sensitive and context-specific interventions, rather than a one-size-fits-all approach.

This review identified inconsistencies in the terminology used to describe stigma, with terms like “affiliate stigma,” “self-stigma,” and “family stigma” being used interchangeably. While this reflects the complexity of stigma, it complicates cross-study comparisons and meta-analyses. To address this issue, we recommend adopting “caregiver affiliate stigma” as a standardized phrase in future research. This would enhance conceptual clarity, improve comparisons across studies, and provide a more unified approach to understanding and addressing the stigma experienced by caregivers.

The application of Bronfenbrenner’s ecological systems theory provides a useful framework for understanding how stigma operates at different societal levels, offering a pathway for targeted interventions:

1. Macrosystem (Cultural Attitudes and Policies): At this level, broader cultural beliefs and systemic policies shape the societal context in which caregivers experience stigma. Interventions at this level might focus on national or regional public awareness campaigns aimed at reducing the stigma associated with caregiving. Policy reforms could include advocacy for caregivers’ mental health services and legal protection that recognizes and mitigate the effects of stigma. Public health campaigns should aim to shift negative perceptions of caregivers and challenge the societal norms that reinforce stigma.
2. Exosystem (Community and Healthcare Settings): The exosystem level involves the indirect impacts of stigma within community and healthcare environments. Interventions at this level could focus on creating stigma-reduction programs within healthcare settings, such as training for healthcare professionals to recognize and address stigma-related issues in relation to caregiving. Community-level interventions, including peer support groups and caregiver-focused outreach programs, could help build supportive networks, reducing the isolation that often exacerbates stigma.
3. Mesosystem (Family Dynamics and Social Networks): The mesosystem represents the intersection of family and social networks, where family dynamics and social relationships may either buffer or exacerbate the effects of stigma. Interventions here could focus on family-based therapies and support groups that help caregivers and family members navigate stigmas together. Strengthening social networks through community engagement, providing respite, and encouraging open communication within families could alleviate caregiver burden and reduce stigma.
4. Microsystem (Individual Experiences and Internalized Stigma): At the microsystem level, the focus is on the individual caregiver’s internalized stigma and daily experiences. Interventions might include individual coping strategies, psychological counseling, and self-empowerment programs. Cognitive behavioral therapy (CBT) and mindfulness training could help caregivers challenge negative self-perceptions and develop resilience against internalized stigma. Self-advocacy training could also empower caregivers to assert their needs in both family and community contexts.

This multilevel approach not only provides a comprehensive framework for understanding how stigma impacts caregivers, but also informs targeted interventions that address stigma in every level of society.

While this review touched on cultural variations in stigma experiences, future research should deepen the exploration of how these differences shape caregivers’ perceptions and coping strategies. Different cultural contexts may require distinct approaches to interventions. For instance, in collectivist cultures, where family reputation is central, family stigma may be more pronounced, necessitating interventions that focus on family dynamics. In contrast, individualistic societies may require interventions targeting self-stigma and personal coping mechanisms. Developing culturally sensitive tools and interventions are critical to effectively support caregivers from diverse backgrounds.

Several limitations of this review should be noted. First, the predominance of cross-sectional studies limits our ability to understand the causal relationships and long-term dynamics of stigma experiences. Longitudinal studies are crucial for capturing how stigma evolves over time and its ongoing impact on caregiver well-being. The focus on English language publications may also have excluded valuable insights from studies published in other languages, limiting the cultural diversity of the findings. Future research should include a broader range of languages to enhance cultural inclusivity.

Additionally, despite Parkinson’s disease being the second most common NDD [8], none of the reviewed studies specifically addressed caregivers of individuals with Parkinson’s disease. This represents a significant gap in the literature, given the unique challenges faced by these caregivers. Future research should focus on this population to provide a more complete understanding of caregiver affiliate stigma in relation to NDDs.

Furthermore, while the application of Bronfenbrenner’s framework offers a robust conceptual structure, future studies should expand on this by investigating how stigma intersects with other social determinants of health, such as socioeconomic status, race, and gender. Research into intersectionality could provide a more nuanced understanding of how different forms of disadvantages compound the effects of caregiver affiliate stigma.

The findings of this review have important implications for both practice and policy. Healthcare providers working with NDD patients and their caregivers should receive training to identify and address the stigma caregivers face. This could involve creating educational modules within healthcare training programs focused on recognizing stigma and developing supportive interventions for caregivers. Providers should also be equipped with resources to guide caregivers toward mental health support services.

From a policy perspective, the review highlights the need for comprehensive strategies that extend beyond the medical management of NDDs to include the social and psychological challenges caregivers face. Policies should address caregiver well-being by funding mental health support, caregiver training, and community outreach programs. Legislative initiatives that offer financial assistance, legal protections, and healthcare benefits to caregivers would also help reduce the stigma by validating their roles and experiences.

## 5. Conclusions

In summary, this scoping review underscores the significant impact of caregiver affiliate stigma on well-being and highlights the need for standardized terminology, culturally sensitive approaches, and multilevel interventions. By addressing stigma across Bronfenbrenner’s ecological model, researchers, policymakers, and practitioners can work together to improve caregiver support systems. These efforts will not only enhance caregiver well-being, but also improve the quality of care for individuals with NDDs, ultimately contributing to better health outcomes for both caregivers and care recipients.

## Figures and Tables

**Table 2:** Key findings from studies on caregiver affiliate stigma in relation to neurodegenerative disorders.

Author	Year of Publication	Key Findings
Bhatt et al.	2022 [46]	Men who strongly identify with traditional male roles and feel judged for caregiving tend to isolate themselves more. Surprisingly, working men struggled more with this than retired ones. Men dealing with their own health issues or who were new to caregiving also tended to withdraw socially. Not knowing exactly what type of dementia their wives had seemed to make men more likely to isolate too.
Brundige	2022 [47]	Higher gender role conflict and social stigma significantly increased self-isolation risk. Contrary to expectations, employed caregivers, especially those working full time and experiencing frequent stigma, were more vulnerable to isolation than retired ones. Caregivers in the early stages (up to 12 months) and those uncertain about their wives’ specific diagnoses showed greater vulnerability. Qualitative data revealed that employment was perceived as an additional burden rather than a respite from caregiving.
Chang et al.	2016 [48]	The Affiliate Stigma Scale demonstrated strong internal consistency, good construct validity with a three-factor structure, and significant concurrent validity with related measures. Rasch's analysis showed a good item fit, with only one potentially problematic item. These findings support the Affiliate Stigma Scale as a valid and reliable tool for measuring affiliate stigma in relation to dementia caregivers, aligning with previous research on the scale.
Chen et al.	2023 [49]	Caregiver burden and affiliate stigma significantly mediated the impact of neuropsychiatric symptoms in people with dementia on caregiver mental health, particularly affecting depression and anxiety levels. Mediation analysis indicated that these symptoms indirectly affect caregiver mental health through both burden and stigma. Additionally, a sequential mediation model suggested that caregiver burden might lead to affiliate stigma, subsequently impacting mental health. The models accounted for a substantial portion of the variance in depression (52.34%) and anxiety (37.72%) among caregivers.
Ellin et al.	2023 [50]	Most caregivers reported low affiliate stigma and moderate to high psychological well-being. A significant negative correlation was found between affiliate stigma and psychological well-being. Female gender and middle-income status were associated with higher affiliate stigma levels. Affiliate stigma emerged as the strongest predictor of caregivers’ psychological well-being, explaining over half of the variance.
Hu et al.	2023 [51]	The results support a theoretical model, where affiliate stigma is negatively associated with quality of life both directly and indirectly through increased caregiving burden and psychological distress. Caregiving burden and psychological distress were found to be sequential mediators in the relationship between affiliate stigma and quality of life.
Jeong et al.	2020 [52]	The results support a moderated mediation model, where coping efficacy mediates the relationship between information cross-checking and coping outcomes. Importantly, this mediation was moderated by affiliate stigma, such that the positive effects of information cross-checking and coping efficacy on outcomes were stronger for caregivers with low affiliate stigma compared to those with high affiliate stigma.
Liu et al.	2014 [53]	There was a significant positive association between perceived stigma and depressive symptoms, both at baseline and over time. This relationship remained significant after controlling for other factors, like ethnicity, location, and severity of dementia symptoms. Additionally, perceived stigma partially mediated the relationship between caregivers’ reactions to dementia-related behaviors and depressive symptoms.
Saffari et al.	2018 [54]	The results show significant indirect effects, with spiritual coping and stigma stress sequentially mediating the associations between patient functioning and caregiver anxiety, depression, burden, and mental quality of life. The mediation models explained substantial variance in caregiver outcomes. Importantly, the order of mediators mattered, with spiritual coping preceding stigma stress in significant models.
Saffari et al.	2019 [38]	The results support the original 3-factor structure (cognitive, affective, and behavioral domains) and demonstrate good psychometric properties, including internal consistency, convergent, and divergent validity. The ASS showed significant correlations with caregiver characteristics like quality of life, depression, anxiety, self-esteem, and social support. A notable finding is the significant negative correlation between affiliate stigma and social support.
Sommers-Spijkerman et al.	2023 [55]	Both patients and caregivers experienced enacted stigma (e.g., social exclusion and staring) and felt stigma (e.g., shame and feeling judged). Patients and caregivers used both concealing and resisting responses to cope with stigma. Factors associated with higher stigma among patients included more bulbar symptoms, intermediate disease stage, younger age, and living without a partner. Common experiences for both patients and caregivers were being stared at and feeling left out
Su and Chang	2020 [56]	High rates of depression (23.7%) and anxiety (37.4%) among caregivers. Male caregivers experienced higher levels of anxiety and care burden related to affiliate stigma compared to females. Caring for younger PWD with lower functional dependence was associated with increased affiliate stigma. The study found a significant positive relationship between caregiver burden and affiliate stigma severity. Caregiver anxiety and overall burden emerged as the strongest predictors of affiliate stigma when accounting for various factors.
Tudose et al.	2017 [57]	Affiliate stigma rates (10.4% to 22.6%) were comparable to international findings. Caregivers reported higher burden levels than in other European studies, particularly in tension and supervision areas. Non-dementia caregivers experienced more tension, while dementia caregivers faced higher supervision burdens. Male caregivers and those caring for younger, more independent patients reported higher affiliate stigma. While most respondents (99.3%) did not perceive professionals’ attitudes as stigmatizing, 43.7% found existing services inadequate for patient needs. Caregiver anxiety and overall burden emerged as the strongest predictors of affiliate stigma.
Van den Bossche and Schoenmakers	2022 [58]	Affiliate stigma significantly affected mental well-being, with women and partners experiencing greater impacts. The duration of dementia diagnosis and caregiver age were also significant factors, with longer duration and older age associated with higher affiliate stigma. Education level had some effect, though the results were mixed.
Velilla et al.	2022 [59]	EOAD caregivers had more socioeconomic risk factors, while FTD caregivers experienced higher levels of family stigma and negative outcomes. Family stigma emerged as the strongest predictor of caregiver outcomes, even after adjusting for other factors. Specifically, higher family stigma was associated with increased caregiver burden and reduced quality of life in terms of energy/fatigue and emotional well-being.
Weisman de Mamani et al.	2017 [60]	Greater perceived stigma was associated with higher levels of expressed emotion by caregivers. Higher expressed emotion was linked to a poorer quality of life for caregivers. The relationship between stigma and quality of life was partially mediated by expressed emotion. Caregivers who felt more stigmatized engaged in more critical and emotionally overinvolved behaviors, which negatively impacted their quality of life.
Werner and AboJabel	2020 [61]	About half of the caregivers experienced affiliate stigma. Lower education, higher courtesy stigma, and lower social support were the main predictors of affiliate stigma. Social support partially mediated the relationship between courtesy and affiliate stigma. Higher education, higher courtesy stigma, and lower social support were associated with greater affiliate stigma. Social support did not moderate the relationship between courtesy and affiliate stigma.
Werner et al.	2011 [39]	The scale assesses three main dimensions: caregiver stigma, lay person stigma, and structural stigma. For caregiver and lay person stigma, the scale confirmed cognitive, emotional, and behavioral aspects. The structural stigma dimension revealed two factors related to caregiver burden and disease severity. Overall, the FS-ADS demonstrated good reliability and validity, explaining large values of variance in each dimension and aligning closely with the theoretical foundations.
Werner et al.	2012 [34]	Caregiver stigma significantly improved prediction of caregiver burden, with shame and decreased caregiving involvement being major contributors. Adult children reported lower stigma levels compared to mental illness caregivers. The lay public dimension of stigma was the most important to caregivers.

## Data Availability

Data sharing is not applicable to this article.

## Supplementary Materials

The following supporting information can be downloaded at: https://www.mdpi.com/article/10.3390/healthcare12191957/s1, Table S1: search strings.
