# Supplementary material for: The Impact of Caregiver Affiliate Stigma on the Psychosocial Well-Being of Caregivers of Individuals with Neurodegenerative Disorders: A Scoping Review
Source: Healthcare (Basel). 2024 Oct 1;12(19):1957. doi: 10.3390/healthcare12191957 (PMC11477483; doi:10.3390/healthcare12191957)
Supplement: Supplementary file 1 [file healthcare-12-01957-s001.zip › healthcare-3180883-supplementary.pdf]

Table S1. Search String Used for PubMed PsycINFO, Embase, CINAHL, and Cochrane Library Databases

Database	Search String
**PubMed**	*("Caregivers"[Mesh] OR "Caregiv*"[Title/Abstract] OR "Spouse Caregivers"[Title/Abstract:~3] OR "Spouse Caregiver"[Title/Abstract:~3] OR "Family Caregiver"[Title/Abstract:~3] OR "Family Caregivers"[Title/Abstract:~3] OR "Informal Caregiver"[Title/Abstract:~3] OR "Informal Caregivers"[Title/Abstract:~3] OR "partner"[Title/Abstract] OR "lay caregiver"[Title/Abstract:~3] OR "lay caregivers"[Title/Abstract:~3] OR "care partner"[Title/Abstract:~3] OR "care partners"[Title/Abstract:~3] OR "family"[Title/Abstract] OR "relative*"[Title/Abstract]) AND ("Social Stigma"[Mesh] OR "stigma"[Title/Abstract] OR "discrimination"[Title/Abstract] OR "prejudice*"[Title/Abstract]) AND ("Neurodegenerative Diseases"[Mesh:NoExp] OR "Neurodegenerative Disease"[Title/Abstract:~5] OR "Neurodegenerative diseases"[Title/Abstract:~5] OR "Neurologic Degenerative Disease"[Title/Abstract:~5] OR "Neurologic Degenerative Diseases"[Title/Abstract:~5] OR "Nervous System Degenerative Disease"[Title/Abstract:~5] OR "Nervous System Degenerative Diseases"[Title/Abstract:~5] OR "Neurodegenerative Disorder"[Title/Abstract:~5] OR "Neurodegenerative Disorder"[Title/Abstract:~5] OR "Degenerative Neurologic Disorder"[Title/Abstract:~5] OR "Degenerative Neurologic Disorders"[Title/Abstract:~5] OR "Huntington Disease"[Mesh] OR "Huntington Chorea"[Title/Abstract:~5] OR "Huntington Disease"[Title/Abstract:~5] OR "Progressive Chorea"[Title/Abstract:~5] OR "Huntington Disease"[Title/Abstract:~5] OR "Tourette Syndrome"[Title/Abstract:~5] OR "Tourettes Syndrome"[Title/Abstract:~5] OR "Tourette Disease"[Title/Abstract:~5] OR "Tourettes Disease"[Title/Abstract:~5] OR "Tourette Disorder"[Title/Abstract:~5] OR "Tourettes Disorder"[Title/Abstract:~5] OR "Chronic Motor and Vocal Tic Disorder"[Title/Abstract] OR "Combined Vocal and Multiple Motor Tic Disorder"[Title/Abstract] OR "Tuberous Sclerosis"[Title/Abstract:~5] OR "Bourneville Phacomatosis"[Title/Abstract:~5] OR "Bourneville Phakomatosis"[Title/Abstract:~5] OR "Bourneville Syndrome"[Title/Abstract:~5] OR "Bourneville Disease"[Title/Abstract:~5] OR "Cerebral Sclerosis"[Title/Abstract:~5] OR "Epiloia"[Title/Abstract] OR "Sclerosis Tuberosa"[Title/Abstract:~5] OR "Tuberose Sclerosis"[Title/Abstract:~5] OR "Adenoma Sebaceum"[Title/Abstract] OR "Motor Neuron Disease"[Mesh] OR "Motor Neuron Disease"[Title/Abstract:~5] OR "Motor Neuron Diseases"[Title/Abstract:~5] OR "Motor System Disease"[Title/Abstract:~5] OR "Motor System Diseases"[Title/Abstract:~5] OR "Lateral Sclerosis"[Title/Abstract:~5] OR "Anterior Horn Cell Disease"[Title/Abstract] OR "Gehrig Disease"[Title/Abstract:~5] OR "Charcot Disease"[Title/Abstract:~5] OR "ALS"[Title/Abstract] OR "Guam Disease"[Title/Abstract:~5] OR "Multiple Sclerosis"[Mesh] OR "Multiple sclerosis"[Title/Abstract] OR "Disseminated Sclerosis"[Title/Abstract] OR "MS"[Title/Abstract])*
PsycInfo	*(Caregivers[MeSH] OR Caregiv*[Title/Abstract] OR Spouse Caregivers[Title/Abstract NEAR/3] OR Spouse Caregiver[Title/Abstract NEAR/3] OR Family Caregiver[Title/Abstract NEAR/3] OR Family Caregivers[Title/Abstract NEAR/3] OR Informal Caregiver[Title/Abstract NEAR/3] OR Informal Caregivers[Title/Abstract NEAR/3] OR partner[Title/Abstract] OR lay caregiver[Title/Abstract NEAR/3] OR lay caregivers[Title/Abstract NEAR/3] OR care partner[Title/Abstract NEAR/3] OR care partners[Title/Abstract NEAR/3] OR family[Title/Abstract] OR relative*[Title/Abstract]) AND (Social Stigma[MeSH] OR stigma[Title/Abstract] OR discrimination[Title/Abstract] OR prejudice*[Title/Abstract]) AND (Neurodegenerative Diseases[MeSH:NoExp] OR Neurodegenerative Disease[Title/Abstract*

	NEAR/5] OR Neurodegenerative diseases[Title/Abstract NEAR/5] OR Neurologic Degenerative Disease[Title/Abstract NEAR/5] OR Neurologic Degenerative Diseases[Title/Abstract NEAR/5] OR Nervous System Degenerative Disease[Title/Abstract NEAR/5] OR Nervous System Degenerative Diseases[Title/Abstract NEAR/5] OR Neurodegenerative Disorder[Title/Abstract NEAR/5] OR Neurodegenerative Disorder[Title/Abstract NEAR/5] OR Degenerative Neurologic Disorder[Title/Abstract NEAR/5] OR Degenerative Neurologic Disorders[Title/Abstract NEAR/5] OR Huntington Disease[MeSH] OR Huntington Chorea[Title/Abstract NEAR/5] OR Huntington Disease[Title/Abstract NEAR/5] OR Progressive Chorea[Title/Abstract NEAR/5] OR Huntington Disease[Title/Abstract NEAR/5] OR Tourette Syndrome[Title/Abstract NEAR/5] OR Tourettes Syndrome[Title/Abstract NEAR/5] OR Tourette Disease[Title/Abstract NEAR/5] OR Tourettes Disease[Title/Abstract NEAR/5] OR Tourette Disorder[Title/Abstract NEAR/5] OR Tourettes Disorder[Title/Abstract NEAR/5] OR Chronic Motor and Vocal Tic Disorder[Title/Abstract] OR Combined Vocal and Multiple Motor Tic Disorder[Title/Abstract] OR Tuberous Sclerosis[Title/Abstract NEAR/5] OR Bourneville Phacomatosis[Title/Abstract NEAR/5] OR Bourneville Phakomatosis[Title/Abstract NEAR/5] OR Bourneville Syndrome[Title/Abstract NEAR/5] OR Bourneville Disease[Title/Abstract NEAR/5] OR Cerebral Sclerosis[Title/Abstract NEAR/5] OR Epiloia[Title/Abstract] OR Sclerosis Tuberosa[Title/Abstract NEAR/5] OR Tuberose Sclerosis[Title/Abstract NEAR/5] OR Adenoma Sebaceum[Title/Abstract] OR Motor Neuron Disease[MeSH] OR Motor Neuron Disease[Title/Abstract NEAR/5] OR Motor Neuron Diseases[Title/Abstract NEAR/5] OR Motor System Disease[Title/Abstract NEAR/5] OR Motor System Diseases[Title/Abstract NEAR/5] OR Lateral Sclerosis[Title/Abstract NEAR/5] OR Anterior Horn Cell Disease[Title/Abstract] OR Gehrig Disease[Title/Abstract NEAR/5] OR Charcot Disease[Title/Abstract NEAR/5] OR ALS[Title/Abstract] OR Guam Disease[Title/Abstract NEAR/5] OR Multiple Sclerosis[MeSH] OR Multiple sclerosis[Title/Abstract] OR Disseminated Sclerosis[Title/Abstract] OR MS[Title/Abstract])
Embase	*(Caregivers[emtree] OR Caregiv*[Title/Abstract] OR Spouse Caregivers[Title/Abstract ADJ 3] OR Spouse Caregiver[Title/Abstract ADJ 3] OR Family Caregiver[Title/Abstract ADJ 3] OR Family Caregivers[Title/Abstract ADJ 3] OR Informal Caregiver[Title/Abstract ADJ 3] OR Informal Caregivers[Title/Abstract ADJ 3] OR partner[Title/Abstract] OR lay caregiver[Title/Abstract ADJ 3] OR lay caregivers[Title/Abstract ADJ 3] OR care partner[Title/Abstract ADJ 3] OR care partners[Title/Abstract ADJ 3] OR family[Title/Abstract] OR relative*[Title/Abstract]) AND (Social Stigma[emtree] OR stigma[Title/Abstract] OR discrimination[Title/Abstract] OR prejudice*[Title/Abstract]) AND (Neurodegenerative Diseases[emtree:noexp] OR Neurodegenerative Disease[Title/Abstract NEAR 5] OR Neurodegenerative diseases[Title/Abstract NEAR 5] OR Neurologic Degenerative Disease[Title/Abstract NEAR 5] OR Neurologic Degenerative Diseases[Title/Abstract NEAR 5] OR Nervous System Degenerative Disease[Title/Abstract NEAR 5] OR Nervous System Degenerative Diseases[Title/Abstract NEAR 5] OR Neurodegenerative Disorder[Title/Abstract NEAR 5] OR Neurodegenerative Disorder[Title/Abstract NEAR 5] OR Degenerative Neurologic Disorder[Title/Abstract NEAR 5] OR Degenerative Neurologic Disorders[Title/Abstract NEAR 5] OR Huntington Disease[emtree] OR Huntington Chorea[Title/Abstract NEAR 5] OR Huntington Disease[Title/Abstract NEAR 5] OR Progressive Chorea[Title/Abstract NEAR 5] OR Huntington Disease[Title/Abstract NEAR 5] OR Tourette Syndrome[Title/Abstract NEAR 5] OR Tourettes Syndrome[Title/Abstract NEAR 5] OR Tourette Disease[Title/Abstract NEAR 5] OR Tourettes Disease[Title/Abstract NEAR 5] OR Tourette Disorder[Title/Abstract NEAR 5] OR Tourettes Disorder[Title/Abstract NEAR 5] OR Chronic Motor and Vocal Tic Disorder[Title/Abstract] OR Combined Vocal and Multiple Motor Tic Disorder[Title/Abstract] OR Tuberous Sclerosis[Title/Abstract NEAR 5] OR Bourneville*

	Phacomatosis[Title/Abstract NEAR 5] OR Bourneville Phakomatosis[Title/Abstract NEAR 5] OR Bourneville Syndrome[Title/Abstract NEAR 5] OR Bourneville Disease[Title/Abstract NEAR 5] OR Cerebral Sclerosis[Title/Abstract NEAR 5] OR Epiloia[Title/Abstract] OR Sclerosis Tuberosa[Title/Abstract NEAR 5] OR Tuberose Sclerosis[Title/Abstract NEAR 5] OR Adenoma Sebaceum[Title/Abstract] OR Motor Neuron Disease[emtree] OR Motor Neuron Disease[Title/Abstract NEAR 5] OR Motor Neuron Diseases[Title/Abstract NEAR 5] OR Motor System Disease[Title/Abstract NEAR 5] OR Motor System Diseases[Title/Abstract NEAR 5] OR Lateral Sclerosis[Title/Abstract NEAR 5] OR Anterior Horn Cell Disease[Title/Abstract] OR Gehrig Disease[Title/Abstract NEAR 5] OR Charcot Disease[Title/Abstract NEAR 5] OR ALS[Title/Abstract] OR Guam Disease[Title/Abstract NEAR 5] OR Multiple Sclerosis[emtree] OR Multiple sclerosis[Title/Abstract] OR Disseminated Sclerosis[Title/Abstract] OR MS[Title/Abstract])
CINAHL	*(Caregivers[Subject Heading] OR Caregiv*[Title/Abstract] OR Spouse Caregivers[Title/Abstract NEAR 3] OR Spouse Caregiver[Title/Abstract NEAR 3] OR Family Caregiver[Title/Abstract NEAR 3] OR Family Caregivers[Title/Abstract NEAR 3] OR Informal Caregiver[Title/Abstract NEAR 3] OR Informal Caregivers[Title/Abstract NEAR 3] OR partner[Title/Abstract] OR lay caregiver[Title/Abstract NEAR 3] OR lay caregivers[Title/Abstract NEAR 3] OR care partner[Title/Abstract NEAR 3] OR care partners[Title/Abstract NEAR 3] OR family[Title/Abstract] OR relative*[Title/Abstract]) AND (Social Stigma[Subject Heading] OR stigma[Title/Abstract] OR discrimination[Title/Abstract] OR prejudice*[Title/Abstract]) AND (Neurodegenerative Diseases[Subject Heading:NoExp] OR Neurodegenerative Disease[Title/Abstract NEAR 5] OR Neurodegenerative diseases[Title/Abstract NEAR 5] OR Neurologic Degenerative Disease[Title/Abstract NEAR 5] OR Neurologic Degenerative Diseases[Title/Abstract NEAR 5] OR Nervous System Degenerative Disease[Title/Abstract NEAR 5] OR Nervous System Degenerative Diseases[Title/Abstract NEAR 5] OR Neurodegenerative Disorder[Title/Abstract NEAR 5] OR Neurodegenerative Disorder[Title/Abstract NEAR 5] OR Degenerative Neurologic Disorder[Title/Abstract NEAR 5] OR Degenerative Neurologic Disorders[Title/Abstract NEAR 5] OR Huntington Disease[Subject Heading] OR Huntington Chorea[Title/Abstract NEAR 5] OR Huntington Disease[Title/Abstract NEAR 5] OR Progressive Chorea[Title/Abstract NEAR 5] OR Huntington Disease[Title/Abstract NEAR 5] OR Tourette Syndrome[Title/Abstract NEAR 5] OR Tourettes Syndrome[Title/Abstract NEAR 5] OR Tourette Disease[Title/Abstract NEAR 5] OR Tourettes Disease[Title/Abstract NEAR 5] OR Tourette Disorder[Title/Abstract NEAR 5] OR Tourettes Disorder[Title/Abstract NEAR 5] OR Chronic Motor and Vocal Tic Disorder[Title/Abstract] OR Combined Vocal and Multiple Motor Tic Disorder[Title/Abstract] OR Tuberous Sclerosis[Subject Heading] OR Bourneville Phacomatosis[Title/Abstract NEAR 5] OR Bourneville Phakomatosis[Title/Abstract NEAR 5] OR Bourneville Syndrome[Title/Abstract NEAR 5] OR Bourneville Disease[Title/Abstract NEAR 5] OR Cerebral Sclerosis[Title/Abstract NEAR 5] OR Epiloia[Title/Abstract] OR Sclerosis Tuberosa[Title/Abstract NEAR 5] OR Tuberose Sclerosis[Title/Abstract NEAR 5] OR Adenoma Sebaceum[Title/Abstract] OR Motor Neuron Disease[Subject Heading] OR Motor Neuron Disease[Title/Abstract NEAR 5] OR Motor Neuron Diseases[Title/Abstract NEAR 5] OR Motor System Disease[Title/Abstract NEAR 5] OR Motor System Diseases[Title/Abstract NEAR 5] OR Lateral Sclerosis[Title/Abstract NEAR 5] OR Anterior Horn Cell Disease[Title/Abstract] OR Gehrig Disease[Title/Abstract NEAR 5] OR Charcot Disease[Title/Abstract NEAR 5] OR ALS[Title/Abstract] OR Guam Disease[Title/Abstract NEAR 5] OR Multiple Sclerosis[Subject*

	Heading] OR Multiple sclerosis[Title/Abstract] OR Disseminated Sclerosis[Title/Abstract] OR MS[Title/Abstract])
Scopus	*(KEYWORDS ("Caregivers" OR "Caregiv*" OR "Spouse Caregivers" OR "Spouse Caregiver" OR "Family Caregiver" OR "Family Caregivers" OR "Informal Caregiver" OR "Informal Caregivers" OR "partner" OR "lay caregiver" OR "lay caregivers" OR "care partner" OR "care partners" OR "family" OR "relative*")) AND (KEYWORDS ("Social Stigma" OR "stigma" OR "discrimination" OR "prejudice*")) AND (KEYWORDS ("Neurodegenerative Diseases" OR "Neurodegenerative Disease" OR "Neurodegenerative diseases" OR "Neurologic Degenerative Disease" OR "Neurologic Degenerative Diseases" OR "Nervous System Degenerative Disease" OR "Nervous System Degenerative Diseases" OR "Neurodegenerative Disorder" OR "Neurodegenerative Disorder" OR "Degenerative Neurologic Disorder" OR "Degenerative Neurologic Disorders" OR "Huntington Disease" OR "Huntington Chorea" OR "Huntington Disease" OR "Progressive Chorea" OR "Huntington Disease" OR "Tourette Syndrome" OR "Tourettes Syndrome" OR "Tourette Disease" OR "Tourettes Disease" OR "Tourette Disorder" OR "Tourettes Disorder" OR "Chronic Motor and Vocal Tic Disorder" OR "Combined Vocal and Multiple Motor Tic Disorder" OR "Tuberous Sclerosis" OR "Bourneville Phacomatosis" OR "Bourneville Phakomatosis" OR "Bourneville Syndrome" OR "Bourneville Disease" OR "Cerebral Sclerosis" OR "Epiloia" OR "Sclerosis Tuberosa" OR "Tuberose Sclerosis" OR "Adenoma Sebaceum" OR "Motor Neuron Disease" OR "Motor Neuron Disease" OR "Motor Neuron Diseases" OR "Motor System Disease" OR "Motor System Diseases" OR "Lateral Sclerosis" OR "Anterior Horn Cell Disease" OR "Gehrig Disease" OR "Charcot Disease" OR "ALS" OR "Guam Disease" OR "Multiple Sclerosis" OR "Multiple sclerosis" OR "Disseminated Sclerosis" OR "MS"))*
